# Deferred Consent in an Acute Stroke Trial from a Patient, Proxy, and Physician Perspective: A Cross-Sectional Survey

**DOI:** 10.1007/s12028-021-01357-3

**Published:** 2021-10-05

**Authors:** Inez Koopman, Dagmar Verbaan, W. Peter Vandertop, Rieke van der Graaf, Erwin J. O. Kompanje, René Post, Bert A. Coert, Martine C. Ploem, Wouter M. Sluis, Féline E. V. Scheijmans, Gabriel J. E. Rinkel, Mervyn D. I. Vergouwen

**Affiliations:** 1 Department of Neurology and Neurosurgery, University Medical Center Utrecht Brain Center, Matthias Van Geuns Building, Room 02.15, University Medical Center Utrecht, Utrecht University, Bolognalaan 2-48, 3584 CJ Utrecht, The Netherlands; 2 Neurosurgical Center Amsterdam, Amsterdam University Medical Centers, Academic Medical Center, Amsterdam, The Netherlands; 3 Department of Medical Humanities, Julius Center for Health Sciences and Primary Care, University Medical Center Utrecht, Utrecht University, Utrecht, The Netherlands; 4 Department of Intensive Care Medicine, Erasmus Medical Center, Rotterdam, The Netherlands; 5 Section of Health Law, Department of Social Medicine, Amsterdam University Medical Centers, University of Amsterdam, Amsterdam, The Netherlands

**Keywords:** Critical care, Subarachnoid hemorrhage, Deferred consent, Ethics, Patients, Proxy, Physician perspective

## Abstract

**Background:**

In some acute care trials, immediate informed consent is not possible, but deferred consent is often considered problematic. We investigated the opinions of patients, proxies, and physicians about deferred consent in an acute stroke trial to gain insight into its acceptability and effects.

**Methods:**

Paper-based surveys were sent to patients who were randomly assigned in the Ultra-early Tranexamic Acid After Subarachnoid Hemorrhage (ULTRA) trial between 2015 and 2018 in two tertiary referral centers and to physicians of centers who agreed or declined to participate. The primary outcome measure was the proportion of respondents who agreed with deferral of consent in the ULTRA trial. Secondary outcomes included respondents’ preferred consent procedure for the ULTRA trial, the effect of deferred consent on trust in physicians and scientific research, and the willingness to participate in future research.

**Results:**

Eighty-nine of 135 (66%) patients or proxies and 20 of 30 (67%) physicians completed the survey. Of these, 82 of 89 (92%) patients or proxies and 14 of 20 (70%) physicians agreed with deferral of consent in the ULTRA trial. When asked for their preferred consent procedure for the ULTRA trial, 31 of 89 (35%) patients or proxies indicated deferred consent, 15 of 89 (17%) preferred immediate informed consent, and 32 of 89 (36%) had no preference. None of the patients’ or proxies’ trust in physicians or scientific research had decreased because of the deferred consent procedure. Willingness to participate in future studies remained the same or increased in 84 of 89 (94%) patients or proxies.

**Conclusions:**

A large majority of the surveyed patients and proxies and a somewhat smaller majority of the surveyed physicians agreed with deferred consent in the ULTRA trial. Deferred consent may enable acute care trials in an acceptable manner without decreasing trust in medicine. Future research should investigate factors facilitating the responsible use of deferred consent, such as in-depth interviews, to study the minority of participants who agreed with deferred consent but still preferred immediate informed consent.

**Supplementary Information:**

The online version contains supplementary material available at 10.1007/s12028-021-01357-3.

## Introduction

In some acute care trials, obtaining informed consent before administration of study medication is not feasible because the therapeutic window is too short to allow ample time for providing, understanding, and considering study information without stress and time pressure [1–4]. Neurological deficits may further impede this decision-making process in acute stroke trials [5]. In a deferred consent procedure, a patient is first enrolled into a study and the patient or proxy is asked in a later phase for consent to use the data for research purposes and to continue the treatment, if applicable. Deferral of consent facilitates unbiased enrollment in emergency research and increases patient recruitment [6–8] but can also compromise patient autonomy [9]. Therefore, specific conditions apply to the use of a deferred consent procedure [10]. Multiple trials have adopted a deferred consent procedure [11–14], but ensuing studies on patients’ and proxies’ opinions about the use of a deferred consent procedure in an intervention study showed conflicting results [15, 16]. In addition, these studies used immediate and deferred consent procedures within one trial, depending on whether deferred consent was legally permitted in the country of interest. In the recently completed Ultra-early Tranexamic Acid After Subarachnoid Hemorrhage (ULTRA) trial, all patients were enrolled using a deferred consent procedure [17, 18]. According to the current legislation in the Netherlands (Medical Research with Human Subjects Act) the deferred consent procedure can be used provided that (1) the study can only be conducted in an emergency situation, (2) consent is impossible to obtain because of the emergency situation, and (3) it involves a “therapeutic” study and participation can potentially benefit the patient. These requirements are further elaborated in a memorandum and a flow chart made by the Dutch Central Ethics Review Committee [19]. The research ethics committee of the Amsterdam University Medical Centers (Amsterdam UMCs) approved the use of deferred consent in the ULTRA trial. When physicians were approached to participate in the ULTRA trial, some declined because they considered the deferred consent procedure unethical. Ethical concerns and questions about the generalizability of previous research led us to investigate the opinions of patients, proxies, and physicians about the use of deferred consent in an acute stroke trial. Such knowledge could help improve acceptability of deferred consent in acute care trials, provided that the majority of patients, proxies, and physicians agree with deferred consent and trust in physicians and scientific research is retained.

## Methods

### Study Design and Population

We conducted a cross-sectional survey study among patients included in the ULTRA trial, their proxies, and physicians of centers who agreed or declined to participate in the ULTRA trial.

The ULTRA trial was a multicenter prospective randomized open-label study in the Netherlands investigating whether ultra-early and short-term administration of the antifibrinolytic agent tranexamic acid (TXA) as an add-on to standard subarachnoid hemorrhage (SAH) management, leads to better functional outcome (NTR3272/NCT02684812) [17, 18]. All patients were recruited using a deferred consent procedure to enable enrollment of a representative sample in the ULTRA trial that included patients with a decreased level of consciousness who did not have a proxy present at bedside. Patients were asked for consent as soon as possible after being randomly assigned unless incapacitated at the time of asking, in which case a proxy was asked for consent. Patients or proxies were asked for consent during day hours after arrival of the patient in the center where the aneurysm was treated. Because TXA was administered for a maximum of 24 h after initiation or until the aneurysm was treated, many patients or proxies were asked for consent after the full dose of TXA had been administered. If the patient or proxy declined participation, the TXA was halted immediately (if applicable) and all study data from the patient was destroyed. From 2013 to 2019, a total of 955 patients were enrolled in the ULTRA trial: 480 patients were randomly assigned to TXA and 475 patients were randomly assigned to the control group [18]. Three patients declined before treatment with TXA was initiated, one proxy declined after TXA had been administered, and two patients or proxies withdrew consent. In the control group, two patients or proxies withdrew consent. Ultra-early, short-term TXA treatment did not improve clinical outcome at 6 months.

For our survey, we sent questionnaires to patients who were randomly assigned and asked for consent for the ULTRA trial at the Amsterdam UMC or the UMC Utrecht between July 2015 and January 2018. Questionnaires were sent between September 2018 and October 2019 (7 months to 4.5 years after enrollment) and had to be filled out by the person who originally provided or declined consent. When the original person was a proxy, the patient was asked to give the survey to the proxy. In the information letter attached to the survey, we clearly specified who provided consent first or declined consent. The survey was sent twice per mail, after which nonrespondents were contacted up to three times by telephone or email. Physicians of centers who agreed or declined to participate were also approached for survey participation. Physicians were contacted twice by mail and twice by email if needed.

Surveys were not sent to patients (1) who had died, (2) who on the ULTRA consent form objected to being contacted for future research, (3) who had nonaneurysmal SAH, (4) whose mailing address or telephone number was unknown or invalid, and (5) who were unable to fill out the survey or give the survey to a proxy at the time of mailing because of cognitive deficits or a language barrier as described in the electronic health record during the 6-month follow-up. A language barrier was defined as insufficient understanding of Dutch to comprehend our questionnaire, e.g., patients who spoke English and had consented on an English version of the ULTRA consent form. We decided not to send surveys to proxies of patients who had died because we considered it potentially hurtful for them to be remembered of the deceased patient. Patients with nonaneurysmal SAH were excluded to avoid confusion in former patients with nonaneurysmal SAH. The rationale of the ULTRA trial can be explained clearly to patients with aneurysmal SAH, as the treatment potentially reduces the risk of rebleeding from an aneurysm. However, to inform patients with nonaneurysmal SAH, one would need to provide more background in the information letter on the pragmatic design of the ULTRA study, which would in turn be superfluous and potentially confusing for patients with nonaneurysmal and aneurysmal SAH. We also did not send questionnaires to (1) physicians who were no longer working at the hospital that agreed or declined to participate in the ULTRA trial, (2) physicians of centers who agreed or declined to participate in the ULTRA trial at a later stage, and (3) physicians who did not respond (e.g., no confirmation or decline) to the invitation to take part in the ULTRA trial as a participating center. Patients, proxies, and physicians who did not want to participate in this survey were asked to return an empty survey.

### Survey Instrument Development

We developed an information letter and six questions to assess the opinions of patients and proxies about the use of deferred consent in the ULTRA trial (Supplemental Data 1). For physicians, an information letter and a four-item survey were developed with similar content but phrased from a physician perspective (Supplemental Data 1). Questions were primarily multiple-choice, closed-ended questions, with one response question that allowed for open ending. For patients and proxies who had declined consent and for physicians of centers who had declined participation, one additional question with an open ending was added to the survey about the reason for not participating in the trial (Supplemental Data 1). Before sending out the questionnaires, the survey was tested in eight patients or proxies and modified to improve clarity and face validity. Content validity was assessed by six authors (DV, WPV, MDIV, GJER, RG, and EJOK). Because the pilot phase resulted in only minor modifications, the answers of the pilot survey were combined with those of the main survey.

### Patients’ and Proxies’ Characteristics

We collected data on the patients’ age and sex and reviewed who provided consent first or declined consent (i.e., the patient or proxy).

### Statistical Analyses

Data were summarized using descriptive statistics.

## Results

**Fig. 1:**
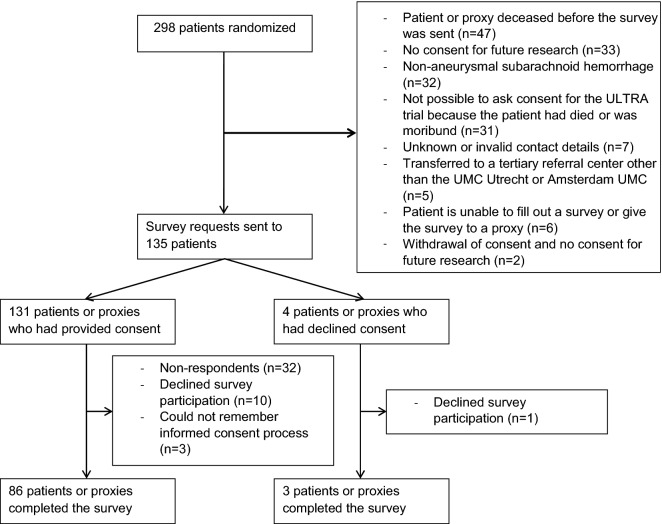
Flowchart of patients or proxies. Amsterdam UMC, Amsterdam University Medical Center, UMCU, University Medical Center Utrecht

**Fig. 2:**
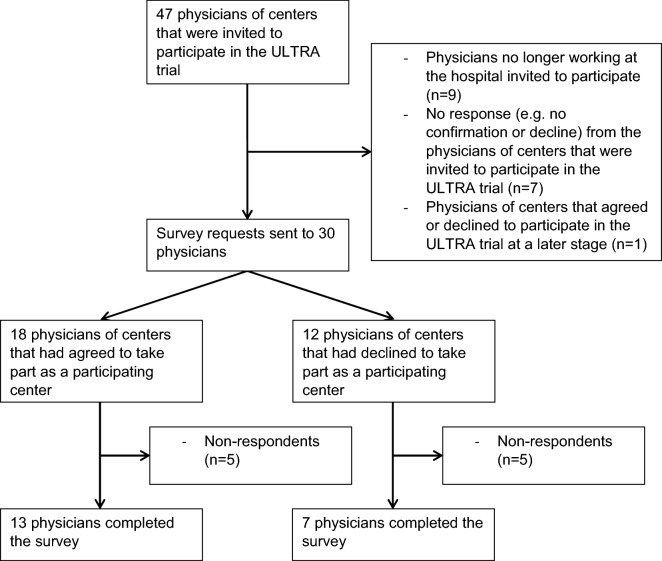
Flowchart physicians. ULTRA, Ultra-early Tranexamic Acid After Subarachnoid Hemorrhage

We sent the questionnaire to 135 patients (14% of all patients enrolled in the ULTRA study), including 4 patients who had declined participation in the ULTRA trial, 18 physicians of centers who had agreed to participate, and 12 physicians of centers who had declined to participate in the ULTRA trial (Figs. 1, 2). In total, 89 of 135 patients or proxies (66% response rate) and 20 of 30 physicians (67% response rate) completed the survey. These included 43 patients and 43 proxies who had provided consent, 2 patients and 1 proxy who had declined consent, 13 local principal investigators of participating centers, and 7 physicians of centers who had declined to participate in the ULTRA trial. Of the 89 patients, 55 (62%) were women and the mean age at the time of randomization was 57 years (range 28–78 years).

### Opinions of Patients or Proxies

Eighty-two of 89 (92%) patients or proxies agreed with the use of deferred consent in the ULTRA trial, 1 of 89 (1%) disapproved the use of deferred consent, and 6 of 89 (7%) did not answer this question (Table 1). None of the patients’ or proxies’ trust in physicians or scientific research had decreased because of the deferred consent procedure. When asked for their preferred consent procedure for the ULTRA trial, 31 of 89 (35%) preferred the use of deferred consent, 8 of 89 (9%) preferred consent for research without explaining its content, 15 of 89 (17%) preferred immediate informed consent, 32 of 89 (36%) had no preference, and 3 of 89 (3%) did not answer this question. Willingness to participate in future research after deferral of consent increased in 25 of 89 (28%) patients or proxies, remained the same in 59 of 89 (66%), decreased in 4 of 89 (5%), and 1 of 89 (1%) patients or proxies did not answer this question. Opinions were similar between patients and proxies (Supplemental Data 2). The two patients and one proxy who had declined consent to participate in the ULTRA study declared this was because of other reasons than the deferred consent procedure.

**Table 1:** Survey results of patients or proxies

Survey questions	Patients or proxies consent (*n* = 86), *n* (%)	Patients or proxies no consent (*n* = 3), *n* (%)	All patients or proxies (*N* = 89), *N* (%)
Do you agree with the use of deferred consent in the ULTRA trial?			
Yes	80 (93)	2 (67)	82 (92)
No	1 (1)	0 (0)	1 (1)
Question not answered	5 (6)	1 (33)	6 (7)
Can you explain why you do not approve the way of asking consent and the procedures for the ULTRA study?			
Informed consent should be asked before trial inclusion	0 (0)	N/A	0 (0)
Other reason(s)	1 (100)	N/A	1 (100)
Could you specify the reason why you did not want to participate in the ULTRA study?			
I never participate in scientific research	N/A	0 (0)	0 (0)
It takes too much time	N/A	1 (33)	1 (33)
Privacy reasons	N/A	0 (0)	0 (0)
The way of asking consent and the procedures for the ULTRA study	N/A	0 (0)	0 (0)
Other reason(s)	N/A	2 (67)	2 (67)
Did the use of deferred consent change your trust in physicians?			
Increase in trust	9 (11)	0 (0)	9 (10)
Remained the same	77 (89)	3 (100)	80 (90)
Decrease in trust	0 (0)	0 (0)	0 (0)
Did the use of deferred consent change your trust in scientific research?			
Increase in trust	19 (22)	1 (33)	20 (22)
Remained the same	67 (78)	2 (67)	69 (78)
Decrease in trust	0 (0)	0 (0)	0 (0)
Which consent procedure do you prefer for the ULTRA trial?			
Deferred consent	30 (35)	1 (33)	31 (35)
Consent for research without explaining its content	8 (9)	0 (0)	8 (9)
Immediate consent	14 (16)	1 (33)	15 (17)
No preference	31 (36)	1 (33)	32 (36)
Question not answered	3 (4)	0 (0)	3 (3)
Did the use of deferred consent in the ULTRA trial change your willingness to participate in future research?			
Increase in willingness	24 (28)	1 (33)	25 (28)
Remained the same	57 (66)	2 (67)	59 (66)
Decrease in willingness	4 (5)	0 (0)	4 (5)
Question not answered	1 (1)	0 (0)	1 (1)

N/A, not applicable, ULTRA, Ultra-early Tranexamic Acid After Subarachnoid Hemorrhage

### Reasons for Disagreement with the Deferred Consent Procedure

The proxy who disagreed with the use of deferred consent had difficulties knowing that a significant other was included in a study. The proxy also reported that the explanation about the study was unclear and that it was a hectic time. The use of deferred consent did not change the proxy’s trust in scientific research, but his or her willingness to participate in future research decreased. The respondent and the patient did, however, consent to continued participation in the ULTRA trial.

### Opinions of Physicians

#### Participating Centers

Twelve of 13 (92%) physicians of centers who agreed to participate felt the patient or proxy would find the use of deferred consent in the ULTRA trial ethically acceptable, and 1 of 13 (8%) did not answer this question (Table 2). All physicians from participating centers (13 of 13, 100%) found the use of deferred consent ethically acceptable. When asked for their preferred consent procedure for the ULTRA trial, 9 of 13 (69%) preferred the use of deferred consent, 3 of 13 (23%) preferred immediate informed consent, and 1 of 13 (8%) had no preference.

**Table 2:** Survey results of physicians

Survey questions	Participating center (*n* = 13), *n* (%)	No participating center (*n* = 7), *n* (%)	All physicians (*N* = 20), *N* (%)
Do you think the patient/proxy considers the use of deferred consent in the ULTRA trial ethically acceptable?			
Acceptable	12 (92)	1 (14)	13 (65)
Not acceptable	0 (0)	3 (43)	3 (15)
Question not answered	1 (8)	3 (43)	4 (20)
Do you consider the consent procedure used in the ULTRA study ethically acceptable?			
Acceptable	13 (100)	1 (14)	14 (70)
Not acceptable	0 (0)	5 (71)	5 (25)
Question not answered	0 (0)	1 (14)	1 (5)
If you consider the consent procedure in the ULTRA study to be unacceptable, can you specify why?			
Informed consent should be asked before trial inclusion	N/A	3 (60)	3 (60)
Informed consent should be asked before trial inclusion in addition to other reason(s)	N/A	1 (20)	1 (20)
Other reason(s)	N/A	1 (20)	1 (20)
Question not answered	N/A	0 (0)	0 (0)
Which consent procedure do you prefer for the ULTRA trial?			
Deferred consent	9 (69)	1 (14)	10 (50)
Consent for research without explaining its content	0 (0)	1 (14)	1 (5)
Immediate consent	3 (23)	3 (43)	6 (30)
No preference	1 (8)	0 (0)	1 (5)
Question not answered	0 (0)	2 (29)	2 (10)
Could you specify the reason why your hospital did not want to take part in the ULTRA study?			
The amount of time required	N/A	0 (0)	0 (0)
The consent procedure	N/A	2 (29)	2 (29)
No financial compensation for included patients	N/A	0 (0)	0 (0)
All of the above	N/A	1 (14)	1 (14)
Other reason(s)	N/A	3 (43)	3 (43)
Question not answered	N/A	1 (14)	1 (14)

N/A, not applicable, ULTRA, Ultra-early Tranexamic Acid After Subarachnoid Hemorrhage

#### Centers Not Participating

One of seven (14%) physicians of centers who had declined to participate in the ULTRA trial felt the patient or proxy would find the use of deferred consent in the ULTRA trial ethically acceptable, 3 of 7 (43%) physicians believed the patient or proxy would find deferral of consent ethically unacceptable, and 3 of 7 (43%) physicians did not answer this question (Table 2). When physicians were asked for their own point of view, 1 of 7 (14%) physicians found the use of deferred consent ethically acceptable, 5 of 7 (71%) physicians found deferred consent ethically unacceptable, and 1 of 7 (14%) physicians did not answer this question. When asked for their preferred consent procedure for the ULTRA trial, 1 of 7 (14%) preferred the use of deferred consent, 1 of 7 (14%) preferred consent for research without explaining its content, 3 of 7 (43%) preferred immediate informed consent, and 2 of 7 (29%) did not answer this question. Of the five physicians who, from their own point of view, considered deferred consent ethically unacceptable, three (60%) physicians indicated that this was because they found that consent should be asked prior to study inclusion, one (20%) questioned the potential benefits of TXA, and one (20%) reported two reasons: this physician did not agree with the logistics of the trial and found that consent should be asked for prior to study inclusion. Three of seven (43%) physicians of centers who had declined to participate in the ULTRA trial declared that this was because of the deferred consent procedure.

## Discussion

A large majority of the surveyed patients and their proxies agreed with the use of a deferred consent procedure in the ULTRA trial. When asked for their preferred consent procedure for the ULTRA trial, most patients or proxies indicated they had “no preference” or preferred “deferred consent.” All patients or proxies indicated that trust in physicians and scientific research had either increased or had remained the same. Patients or proxies also remained willing to participate in future research. None of the patients or proxies reported ethical concerns about the use of deferred consent in the ULTRA trial.

Although also most physicians agreed with the use of deferred consent, the proportion of physicians who were against its use was larger than that of patients or proxies. However, the restricted therapeutic window in the ULTRA trial precludes ample time to come to a well-informed decision about trial participation, meaning that the ULTRA trial cannot be conducted if consent is required before administration of study medication.

Although most patients, proxies and physicians agreed with the use of deferred consent, some preferred another procedure. Our results show that patients, proxies, and physicians can agree with deferred consent, but at the same time they can have no preference or a different preference for the consent model used in the ULTRA trial. The reason for this discrepancy is unclear from our study. In-depth interviews might be a better-suitable instrument to investigate such a question because knowledge about ethics regulations and a deep understanding of the different consent procedures are required to answer this question. Although an explanation about the different consent procedures and the implications of its use were included in the survey, this information may have been insufficient.

Several studies have investigated support from patients, proxies, and the general public for exception from informed consent, in which no consent at all, also not deferred consent, is asked for research in an emergency setting [20–25]. Estimates of support ranged 35–84% and depended on factors such as population, type of disease (e.g., trauma or neurological disease), and risks associated with the intervention [21–24]. Exception from informed consent obliges researchers to inform the patient, proxy, or family member about the patient’s inclusion and the clinical study at the earliest feasible opportunity [26]. The patient’s participation may be discontinued at any time. However, a signature on the consent form, such as required with deferred consent, is not obliged. The results of these previous studies can, therefore, not be generalized to trials using a deferred consent procedure.

In the two previous studies on deferred consent in intervention trials, patients and proxies generally agreed with deferral of consent in the study investigating treatment with medication (intensive maintenance of blood glucose levels with insulin), but mostly disagreed in the study researching a more invasive treatment (endovascular thrombectomy) [15, 16]. Our study, which also investigated treatment with medication, found that a large majority agreed with the use of deferred consent. The invasiveness of the intervention tested may, in part, determine patients’ and proxies’ opinions on deferred consent. Regarding the time points of survey administration, surveys were conducted during the trial in the study in which patients disagreed with deferral of consent [16]. Both our study and the other study, in which patients agreed with deferral of consent, conducted the surveys after trial completion [15]. Nevertheless, we do not expect the different time points of survey administration to explain the conflicting results because we would assume that patients’ and proxies’ opinions about the use of a deferred consent procedure do not change over time. The latter hypothesis is supported by previous results from the study in which patients disagreed with deferred consent: similar opinions were found 4 days after ictus versus 90 days after ictus [16]. Although administration of the survey after trial completion could affect recall of the consent process, in our study only three patients could not remember the consent process.

In the information letter attached to our survey, we explained why deferred consent was used, that the study medication was considered safe, and that the use of deferred consent had been approved by the appropriate ethics committee. This explanation was more extensive than in the endovascular intervention study, in which the majority of patients disagreed with deferral of consent [16]. Based on the experience of authors who conducted deferred consent conversations, we would hypothesize that a better understanding of why a deferred consent procedure is used in emergency research, and under which conditions it is allowed, might lead to more patients, proxies, and physicians to agree with the use of a deferred consent procedure in acute care trials.

A strength of our study is that the opinions of patients, proxies, and physicians were asked, also those who declined participation. In addition, we first conducted a pilot survey to improve clarity and face validity of the survey, as there are no validated surveys available on deferred consent. We incorporated one or two open-ended questions in our survey to provide further insight into responses to the multiple-choice, closed-ended questions.

Some limitations need to be addressed. First, recall bias could have influenced our results because we asked our respondents to reflect on events that took place between 2015 and 2018. To decrease recall bias, we attached an information letter explaining the rationale of the ULTRA trial, deferred consent, and why it was used. However, the phrasing and information provided in the information letter may have influenced the respondents’ answers. Second, the survey was administered at two sites of the ULTRA trial. These sites enrolled the majority of patients in the ULTRA trial and provided a large enough sample for the study. The baseline characteristics and outcomes in the two participating centers were comparable with those of the other centers. Nevertheless, selection bias cannot be ruled out. Third, survey requests were not sent to certain groups of patients (e.g., to proxies of patients who had died and patients who declined consent for future research). Exclusion of these groups could have affected our results, as these groups may be more negative toward a deferred consent procedure. Fourth, although our survey had a good overall response rate, 34% of the patients or proxies and 33% of the physicians did not fill out the survey despite multiple reminders. We can therefore not exclude a nonresponse bias. Nevertheless, even if all patients or proxies who did not fill out the survey would disapprove deferral of consent, the majority of our sample would still agree with the use deferred consent in the ULTRA trial. In addition, the sample of patients or proxies who declined participation in the ULTRA trial and physicians is small and our ability to draw conclusions from their opinions is limited. Lastly, it remains unclear from our survey whether the differences in results between patients or proxies and physicians may be explained by the different roles of both groups.

## Conclusions

Our study found that the use of a deferred consent procedure in the ULTRA trial is accepted by a large majority of the surveyed patients, proxies, and physicians. Deferred consent may enable acute care trials in an acceptable manner without decreasing trust in physicians or scientific research. Future research should investigate factors that facilitate responsible use, both legally and ethically, of a deferred consent procedure in acute intervention trials.

## Supplementary Information

Below is the link to the electronic supplementary material.

Supplementary file1 (DOCX 36 KB)
