# Supplementary material for: Deferred Consent in an Acute Stroke Trial from a Patient, Proxy, and Physician Perspective: A Cross-Sectional Survey
Source: Neurocrit Care. 2021 Oct 5;36(2):621–9. doi: 10.1007/s12028-021-01357-3 (PMC8964548; doi:10.1007/s12028-021-01357-3)
Supplement: Supplementary file 1 — Supplementary file1 (DOCX 36 KB) [file 12028_2021_1357_MOESM1_ESM.docx]

**Supplemental Material
Deferred consent in an acute stroke trial from a patient, proxy, and physician perspective: a cross-sectional survey**Inez Koopman, MD; Dagmar Verbaan, PhD; W. Peter Vandertop, MD PhD^;^ Rieke van der Graaf, PhD; Erwin J.O. Kompanje, PhD; René Post, MD; Bert A. Coert, MD PhD; Martine C. Ploem, PhD; Wouter M. Sluis, MD; Féline E.V. Scheijmans, MD; Gabriel J.E. Rinkel, MD FRCP(E); Mervyn D.I. Vergouwen, MD PhD

**Supplemental data 1. Information letters and surveys** **p. 2-24**
Translation of the information letters and surveys p. 2
Information letter for patients p. 3-8
Information letter for proxies p. 9-15
Information letter for neurologists/neurosurgeons p. 16-21
Survey for patients who provided consent for the ULTRA trial p. 22-24
Survey for patients who declined consent for the ULTRA trial p. 25-28
Survey for proxies who provided consent for the ULTRA trial p. 29-31
Survey for proxies who declined consent for the ULTRA trial p. 32-35
Survey for physicians of centers that agreed to participate in the ULTRA trial p. 36-37
Survey for physicians of centers that declined to participate in the ULTRA trial p. 38-40
**Supplemental data 2. Survey results patients or proxies**  **p. 41-43**

*Translation of the information letters and surveys*Forward translation of the information letters and surveys from its original language (Dutch) into English was performed by one of the authors (IK). Subsequently, the letters and surveys was back-translated by another author (GJER). Discrepancies between the forward- and backward translation were discussed in an expert committee (IK, GJER, MDIV) until consensus about the English translation was reached.

Information letter for patients

**Information letter about the way of asking consent and the procedures of the ULTRA study**

Dear Sir/Madame,

You are receiving this letter because you have been approached for a scientific study (the ULTRA study) during your in-hospital stay due to a brain hemorrhage. We kindly ask you to participate in a survey study to investigate how you have experienced the way of asking consent and the procedures for the ULTRA study. Information about the survey study can be found in this information letter and appendix 1. Participation in this survey study is voluntary. It will take approximately 5 minutes to fill out the questionnaire. Thank you in advance for your cooperation.

**1. What is the aim of this survey study?**You have had a brain hemorrhage (subarachnoid hemorrhage/SAH) for which you have been hospitalized. During you hospital stay, you were asked to participate in a research study, namely the Ultra-Early Tranexamic Acid After Subarachnoid Hemorrhage (ULTRA) study. We would like to investigate with a questionnaire how you have experienced the way of asking consent and the procedures for the ULTRA study.

**2. ULTRA study**

A brain hemorrhage is usually caused by the rupture of a bulge (aneurysm) in an artery. If a hemorrhage occurred, there is high probability that the bulge will rupture again shortly afterwards. A new hemorrhage worsens the prognosis. The ULTRA study investigates whether early and short-term administration of a particular drug reduces the risk of a new hemorrhage and thereby improves prognosis.

**3. Way of asking consent in most studies**

In most studies, you will first receive an explanation about the research study before you are asked whether you would like to participate in the research study. The drug, which is being investigated, will only be administered after you have given permission to participate in the study.

**4. Way of asking consent in the ULTRA study**

A different way of asking consent and procedure have been used in the ULTRA study. First, lots were drawn to determine whether you were given the study medication. If you drew lots for the study medication, it was administered to you. You received an explanation about the study and were asked whether you would want to participate only after the start of the study medication. If you did not receive the study medication, the explanation about the study and asking for consent were also carried out after lots were drawn. A research ethics committee approved the way of asking consent, the procedures, and the conduct of the ULTRA study.

**5. Why this way of asking consent and procedure?**

The reason why a different way of asking consent and procedure were used in the ULTRA study is because the treatment investigated should be given as soon as possible after the brain hemorrhage. The risk of a new hemorrhage is highest directly after the first hemorrhage. Time for an explanation about the research study and reflection are lacking, because a lot of valuable time would be lost during a time period in which the risk of a new hemorrhage is high. In addition, we know that the study drug is safe. You received an explanation about the study at a later stage, but as soon as possible. If you did not want to participate in the study, the administration of the study drug was halted immediately (if applicable) and your medical data were not used for the study.

**6. Participation in the survey study**

You decide whether you would like to participate in this survey study. If you would like to participate, we ask you to fill out the questionnaire and return it with the stamped return envelope. If you decide not to participate in this research study, you can return an empty questionnaire. If such an empty questionnaire is returned, you will not be approached again. You do not have to give a reason for not filling out the questionnaire, this will not affect your treatment. Additional information about this survey study can be found in appendix I. Should you have any questions, please feel free to contact the researchers (appendix 2).

We would like to thank you for your time and hope that you are willing to fill out the questionnaire.

Kind regards,

I. Koopman, physician and PhD candidate, UMC Utrecht

Dr. M.D.I. Vergouwen, neurologist, UMC Utrecht

Prof. G.J.E. Rinkel, neurologist, UMC Utrecht

Prof. W.P. Vandertop, neurosurgeon and principal investigator ULTRA, Amsterdam UMC
Dr. D. Verbaan, clinical epidemiologist and principal investigator ULTRA, Amsterdam UMC

Dr. R. Van der Graaf, assistant professor medical ethics, UMC Utrecht

**Appendix 1: Additional information on the survey study**

**1. What are the possible advantages and disadvantages of participating in this research study?**

If you participate in this research study, there are no benefits for yourself. However, it could provide useful information about the way of asking consent for research in emergency situations. A disadvantage of this study is that it will cost you extra time.

**2. What happens if the research study has ended?**

After the research study has ended, the results will be examined. If you are interested, we can inform you about the results on group-level. You can indicate this on the questionnaire.

**3. What happens with your data?**

Your data will be stored with a code. Only the researchers have access to this code and can therefore read back what you filled out.

**4. Are there additional costs/is there compensation when you decide to participate in this research study?**

You will not receive a compensation and there are no costs involved for this survey study.

**5. How to act if you have complaints?**

Should you be dissatisfied about the course of events of this survey study and you would like to file a complaint, please contact patient services. They are available via the following phone number: XXX-XXXXXX.

If you are dissatisfied about the way of asking consent and the procedure of the ULTRA study, you can contact the investigators (appendix 2). We can then discuss this with you.

**6. Conflict of interest and funding of the research study**

The researchers declare that they do not hold any positions that may lead to conflicting interest for this study. The research study is not funded.

**Appendix II: Contact details**

University Medical Center Utrecht

Physician and PhD candidate: I. Koopman

Phone number: XXX-XXXXXXX

E-mail: XX

Principal investigator: Dr. M.D.I. Vergouwen, neurologist

Phone number XXX-XXXXXXX

E-mail: XX

Information letter for proxies

**Information letter about the way of asking consent and the procedures of the ULTRA study**

Dear Sir/Madame,

You are receiving this letter because you have been approached for a scientific study (the ULTRA study) during the in-hospital stay of your family member or partner due to a brain hemorrhage. You were asked if you wanted to provide consent for your family member or partner to participate in the ULTRA study because your family member or partner was not able to provide consent at that moment. We would like to investigate with a questionnaire how you have experienced the way of asking consent and the procedure. We kindly ask you to participate in a survey study. Information about the survey study can be found in this information letter and appendix 1. Participation in this survey study is voluntary. It will take approximately 5 minutes to fill out the questionnaire. Thank you in advance for your cooperation.

**1. What is the aim of this survey study?**You family member or partner had a brain hemorrhage (subarachnoid hemorrhage/SAH) for which he/she was admitted to the hospital. During the in-hospital stay, you were asked if you wanted to provide consent for your family member or partner to participate in a research study, namely the Ultra-Early Tranexamic Acid After Subarachnoid Hemorrhage (ULTRA) study. You were asked this question because your family member or partner was unable to decide about participation in a research study. We would like to investigate with a questionnaire how you have experienced the way of asking consent and the procedures for the ULTRA study.

**2. ULTRA study**

A brain hemorrhage is usually caused by the rupture of a bulge (aneurysm) in an artery. If a hemorrhage occurred, there is high probability that the bulge will rupture again shortly afterwards. A new hemorrhage worsens the prognosis. The ULTRA study investigates whether early and short-term administration of a particular drug reduces the risk of a new hemorrhage and thereby improves prognosis.

**3. Way of asking consent in most studies**

In most studies, you will first receive an explanation about the research study before you are asked whether you would like to participate in the research study. The drug, which is being investigated, will only be administered after you have given permission for your family member or partner to participate in the research study

**4. Way of asking consent and procedure in the ULTRA study**

A different way of asking consent and procedure have been used in the ULTRA study. First, lots were drawn to determine who received the study medication. If your family member or partner drew lots for the study medication, he/she was given the study medication. You received an explanation about the study and were asked whether you would want to provide consent for your family member or partner to participate in the research study only after the start of the study medication. If your family member or partner did not receive the study medication, the explanation about the study and asking for consent were also carried out after lots were drawn. A research ethics committee approved the way of asking consent, the procedures, and the conduct of the ULTRA study.

**5. Why this way of asking consent and procedure?**

The reason why a different way of asking consent and procedure were used in the ULTRA study is because the treatment investigated should be given as soon as possible after the brain hemorrhage. The risk of a new hemorrhage is highest directly after the first hemorrhage. Time for an explanation about the research study and reflection are lacking, because a lot of valuable time would be lost during a time period in which the risk of a new hemorrhage is high. In addition, we know that the study drug is safe. You received an explanation about the study at a later stage, but as soon as possible. If you did not want to participate in the study, the administration of the study drug was halted immediately (if applicable) and your medical data were not used for the study.

**6. Participation in the survey study**

You decide whether you would like to participate in this survey study. If you would like to participate, we ask you to fill out the questionnaire and return it with the stamped return envelope. If you decide not to participate in this research study, you can return an empty questionnaire. If such an empty questionnaire is returned, you will not be approached again. You do not have to give a reason for not filling out the questionnaire, this will not affect the treatment of your family member or partner. Additional information about this survey study can be found in appendix I. Should you have any questions, please feel free to contact the researchers (appendix 2).

We would like to thank you for your time and hope that you are willing to fill out the questionnaire.

Kind regards,

I. Koopman, physician and PhD candidate, UMC Utrecht

Dr. M.D.I. Vergouwen, neurologist, UMC Utrecht

Prof. G.J.E. Rinkel, neurologist, UMC Utrecht

Prof. W.P. Vandertop, neurosurgeon and principal investigator ULTRA, Amsterdam UMC
Dr. D. Verbaan, clinical epidemiologist and principal investigator ULTRA, Amsterdam UMC

Dr. R. Van der Graaf, assistant professor medical ethics, UMC Utrecht

**Appendix 1: Additional information on the survey study**

**1. What are the possible advantages and disadvantages of participating in this research study?**

If you participate in this research study, there are no benefits for yourself. However, it could provide useful information about the way of asking consent for research in emergency situations. A disadvantage of this study is that it will cost you extra time.

**2. What happens if the research study has ended?**

After the research study has ended, the results will be examined. If you are interested, we can inform you about the results on group-level. You can indicate this on the questionnaire.

**3. What happens with your data?**

Your data will be stored with a code. Only the researchers have access to this code and can therefore read back what you filled out.

**4. Are there additional costs/is there compensation when you decide to participate in this research study?**

You will not receive a compensation and there are no costs involved for this survey study.

**5. How to act if you have complaints?**

Should you be dissatisfied about the course of events of this survey study and you would like to file a complaint, please contact patient services. They are available via the following phone number: XXX-XXXXXX.

If you are dissatisfied about the way of asking consent and the procedure of the ULTRA study, you can contact the investigators (appendix 2). We can then discuss this with you.

**6. Conflict of interest and funding of the research study**

The researchers declare that they do not hold any positions that may lead to conflicting interest for this study. The research study is not funded.

**Appendix II: Contact details**

University Medical Center Utrecht

Physician and PhD candidate: I. Koopman

Phone number: XXX-XXXXXXX

E-mail: XX

Principal investigator: Dr. M.D.I. Vergouwen, neurologist

Phone number XXX-XXXXXXX

E-mail: XX

Information letter for neurologists/neurosurgeons

**Information letter about the way of asking consent and the procedures of the ULTRA study**

Dear Sir/Madame,

We kindly ask you to participate in a survey study. You are receiving this letter because your department of neurology/neurosurgery has been approached in the past to participate in the Early Tranexamic Acid After Subarachnoid Hemorrhage (ULTRA) study. With a questionnaire, we would like to investigate your opinion as a neurologist/neurosurgeon about the way of asking consent and procedure for the ULTRA study. Information about the survey study can be found in this information letter and appendix 1. Participation in this survey study is voluntary. It will take approximately 5 minutes to fill out the questionnaire. Thank you in advance for your cooperation.

**1. What is the aim of this survey study?**

We would like to investigate what your opinion as a neurologist/neurosurgeon is about the way of asking consent and the procedure for the ULTRA study.

**2. ULTRA study**

The ULTRA study investigates whether short-term administration of tranexamic acid reduces the risk of rebleeding and improves prognosis in patients with an aneurysmal subarachnoid hemorrhage. The ULTRA study is coordinated by researchers from the Academic Medical Centrum (AMC) in Amsterdam. The University Medical Center Utrecht (UMCU) participates in this research study.

**3. Consent statement**

In most studies, it is common for the patient or proxy to be informed about the content, aim and risks of the research study before consent is asked (informed consent). The study medication can only be given after the consent form is signed.

**4. Deferred consent**

A deferred consent procedure is used in the ULTRA study. First, lots were drawn to determine who received the study medication. If lots were drawn for the study medication, it was administered. Only after starting the study medication, but as soon as possible, the patient or proxy was informed about the research study and asked for consent for further participation and use of medical data. For patients in the care as usual group (without study medication), the explanation about the study and asking for consent were also carried out after lots were drawn. If the patient or proxy did not provide consent for the research study, the administration of the study drug was halted immediately (if applicable) and the medical data were not used for the study. The research ethics committee of the AMC in Amsterdam approved the deferred consent procedure and the research study. All of the due diligence procedures for the approval of the ULTRA study have been followed.

**5. Why deferred consent**

The reason why the ULTRA study uses a deferred consent procedure is because tranexamic acid should be given as soon as possible after the subarachnoid hemorrhage to achieve a potential therapeutic effect. The risk of a new hemorrhage is highest directly after the first hemorrhage. Time for an explanation about the research study and reflection are lacking, because a lot of valuable time would be lost during a time period in which the risk of a new hemorrhage is high. In addition, we know that the study drug is safe.

**6. Participation in the survey study**

You decide whether you would like to participate in this survey study. If you would like to participate, we ask you to fill out the questionnaire and return it with the stamped return envelope. If you decide not to participate in this research study, you can return an empty questionnaire. If such an empty questionnaire is returned, you will not be approached again. Additional information about this survey study can be found in appendix I. Should you have any questions, please feel free to contact the researchers (appendix 2).

We would like to thank you for your time and hope that you are willing to fill out the questionnaire.

Kind regards,

I. Koopman, physician and PhD candidate, UMC Utrecht

Dr. M.D.I. Vergouwen, neurologist, UMC Utrecht

Prof. G.J.E. Rinkel, neurologist, UMC Utrecht

Prof. W.P. Vandertop, neurosurgeon and principal investigator ULTRA, Amsterdam UMC
Dr. D. Verbaan, clinical epidemiologist and principal investigator ULTRA, Amsterdam UMC

Dr. R. Van der Graaf, assistant professor medical ethics, UMC Utrecht

**Appendix 1: Additional information on the survey study**

**1. What are the possible advantages and disadvantages of participating in this research study?**

If you participate in this research study, there are no benefits for yourself. However, it could provide useful information about the way of asking consent for research in emergency situations. A disadvantage of this study is that it will cost you extra time.

**2. What happens if the research study has ended?**

After the research study has ended, the results will be examined. If you are interested, we can inform you about the results on group-level. You can indicate this on the questionnaire.

**3. What happens with your data?**

Your data will be stored with a code. Only the researchers have access to this code and can therefore read back what you filled out.

**4. Are there additional costs/is there compensation when you decide to participate in this research study?**

You will not receive a compensation and there are no costs involved for this survey study.

**5. How to act if you have complaints?**

Should you be dissatisfied about the course of events of this survey study and you would like to file a complaint, please contact patient services. They are available via the following phone number: XXX-XXXXXX.

**6. Conflict of interest and funding of the research study**

The researchers declare that they do not hold any positions that may lead to conflicting interest for this study. The research study is not funded.

**Appendix II: Contact details**

University Medical Center Utrecht

Physician and PhD candidate: I. Koopman

Phone number: XXX-XXXXXXX

E-mail: XX

Principal investigator: Dr. M.D.I. Vergouwen, neurologist

Phone number XXX-XXXXXXX

E-mail: XX

Survey for patients who provided consent for the ULTRA trial

**Survey about the ULTRA study**

1. As was explained in the information letter, the way of asking consent and the procedures for the ULTRA study are different from other studies. Only after administration of the study medication, you received an explanation about the study. At the time of explanation, you were asked for consent to use your data for research purposes. If you did not receive the study medication, the explanation about the study and asking for consent also took place after drawing of lots. This was done because the treatment investigated in the ULTRA study has to be administered as soon as possible after the brain hemorrhage. Do you agree with the way of asking consent and the procedures for the ULTRA study?
o Yes. You can continue with question 3.
o No. You can continue with question 2.

2. Why do you not agree with the way of asking consent and the procedures for the ULTRA study? If you would like to, you can check multiple answers.
o Your opinion is that you should receive an explanation about the study before you consent to participate in the study. This should also be done if this means it is practically not possible to carry out the study and that the possible benefit to you no longer applies
o Other reason(s), namely:

……………………………………………………………………………………………………………………………………….…………………………………………………………………………………………………………………………………………….…………………………………………………………………………………………………………………………………

3. Did the way of asking consent and the procedures for the ULTRA study change your trust in physicians?
o I have more trust in physicians
o My trust in physicians remained the same
o My trust in physicians decreased

4. Did the way of asking consent and the procedures for the ULTRA study change your trust in scientific research?
o I have more trust in scientific research
o My trust in scientific research remained the same
o My trust in scientific research decreased

5. Which way of asking consent and which procedures do you prefer for the ULTRA study?
o An explanation about the study is provided and afterwards consent for participation in the study is asked. Treatment with study medication can only begin when consent is provided. Because of the possibility of a cooling-off period, the study medication will be administered at a later point in time, while the risk of a new bleeding is highest just after the first bleeding.
o Consent is asked for participation in a scientific study without any additional explanation about what the study exactly entails. The explanation about the study will be given after the study has been completed.
o Lots are drawn and if the outcome is treatment, the study medication will be administered. Only after administration of the study medication, you will receive an explanation about the study and are asked for consent to continue participation.
o It does not matter to you which way of consent and procedures are used for the ULTRA study.

6. Did the way of asking consent and the procedures for the ULTRA study change your opinion to participate in future scientific research?
o Yes, I am more willing to participate in future scientific research
o Yes, I am less willing to participate in future scientific research
o No, my willingness to participate in future scientific research remained the same

7. Would you like us to inform you about the results (at group level) of this survey?
o Yes
o No

Thank you for filling out this survey. For questions or remarks, you can contact the researchers (Appendix II).

Survey for patients who declined consent for the ULTRA trial

**Survey about the ULTRA study**

1. What was the reason you did not want to participate in the ULTRA study? You do not need to answer this question. If you do not answer this question, there will be no consequences for your future treatment.
o I never participate in scientific medical research
o It takes too much time
o Privacy reasons
o The way of asking consent and the procedures for the ULTRA study
o Other reason(s), namely: ………………………………………………………………………………………………………………………………………

2. As was explained in the information letter, the way of asking consent and the procedures for the ULTRA study are different from other studies. Only after administration of the study medication, you received an explanation about the study. At the time of explanation, you were asked for consent to use your data for research purposes. If you did not receive the study medication, the explanation about the study and asking for consent also took place after drawing of lots. This was done because the treatment investigated in the ULTRA study has to be administered as soon as possible after the brain hemorrhage. Do you agree with the way of asking consent and the procedures for the ULTRA study?
o Yes. You can continue with question 4.
o No. You can continue with question 3.

3. Why do you not agree with the way of asking consent and the procedures for the ULTRA study? If you would like to, you can check multiple answers.
o Your opinion is that you should receive an explanation about the study before you consent to participate in the study. This should also be done if this means it is practically not possible to carry out the study and that the possible benefit to you no longer applies
o Other reason(s), namely:

……………………………………………………………………………………………………………………………………….…………………………………………………………………………………………………………………………………………….…………………………………………………………………………………………………………………………………

4. Did the way of asking consent and the procedures for the ULTRA study change your trust in physicians?
o I have more trust in physicians
o My trust in physicians remained the same
o My trust in physicians decreased

5. Did the way of asking consent and the procedures for the ULTRA study change your trust in scientific research?
o I have more trust in scientific research
o My trust in scientific research remained the same
o My trust in scientific research decreased

6. Which way of asking consent and which procedures do you prefer for the ULTRA study?
o An explanation about the study is provided and afterwards consent for participation in the study is asked. Treatment with study medication can only begin when consent is provided. Because of the possibility of a cooling-off period, the study medication will be administered at a later point in time, while the risk of a new bleeding is highest just after the first bleeding.
o Consent is asked for participation in a scientific study without any additional explanation about what the study exactly entails. The explanation about the study will be given after the study has been completed.
o Lots are drawn and if the outcome is treatment, the study medication will be administered. Only after administration of the study medication, you will receive an explanation about the study and are asked for consent to continue participation.
o It does not matter to you which way of consent and procedures are used for the ULTRA study.

7. Did the way of asking consent and the procedures for the ULTRA study change your opinion to participate in future scientific research?
o Yes, I am more willing to participate in future scientific research
o Yes, I am less willing to participate in future scientific research
o No, my willingness to participate in future scientific research remained the same

8. Would you like us to inform you about the results (at group level) of this survey?
o Yes
o No
Thank you for filling out this survey. For questions or remarks, you can contact the researchers (Appendix II).

Survey for proxies who provided consent for the ULTRA trial

**Survey about the ULTRA study**

1. As was explained in the information letter, the way of asking consent and the procedures for the ULTRA study are different from other studies. Only after administration of the study medication to your family member or significant other, you received an explanation about the study. At the time of explanation, you were asked for consent to use the data for research purposes. If your family member or significant other did not receive the study medication, the explanation about the study and asking for consent also took place after drawing of lots. This was done because the treatment investigated in the ULTRA study has to be administered as soon as possible after the brain hemorrhage. Do you agree with the way of asking consent and the procedures for the ULTRA study?
o Yes. You can continue with question 3.
o No. You can continue with question 2.

2. Why do you not agree with the way of asking consent and the procedures for the ULTRA study? If you would like to, you can check multiple answers.
o Your opinion is that you should receive an explanation about the study before you consent for your family member or significant other to participate in the study. This should also be done if this means it is practically not possible to carry out the study and that the possible benefit to your family member or significant other no longer applies
o Other reason(s), namely:
……………………………………………………………………………………………………………………………………….…………………………………………………………………………………………………………………………………………….…………………………………………………………………………………………………………………………………

3. Did the way of asking consent and the procedures for the ULTRA study change your trust in physicians?
o I have more trust in physicians
o My trust in physicians remained the same
o My trust in physicians decreased

4. Did the way of asking consent and the procedures for the ULTRA study change your trust in scientific research?
o I have more trust in scientific research
o My trust in scientific research remained the same
o My trust in scientific research decreased

5. Which way of asking consent and which procedures do you prefer for the ULTRA study?
o An explanation about the study is provided and afterwards consent for participation in the study is asked. Treatment with study medication can only begin when consent is provided. Because of the possibility of a cooling-off period, the study medication will be administered at a later point in time, while the risk of a new bleeding is highest just after the first bleeding.
o Consent is asked for participation in a scientific study without any additional explanation about what the exactly study entails. The explanation about the study will be given after the study has been completed.
o Lots are drawn and if the outcome is treatment, the study medication will be administered. Only after administration of the study medication, you will receive an explanation about the study and are asked for consent to continue participation.
o It does not matter to you which way of consent and procedures are used for the ULTRA study.

6. Did the way of asking consent and the procedures for the ULTRA study change your opinion to participate in future scientific research?
o Yes, I am more willing to participate in future scientific research
o Yes, I am less willing to participate in future scientific research
o No, my willingness to participate in future scientific research remained the same

7. Would you like us to inform you about the results (at group level) of this survey?
o Yes
o No

Thank you for filling out this survey. For questions or remarks, you can contact the researchers (Appendix II).

Survey for proxies who declined consent for the ULTRA trial

**Survey about the ULTRA study**

1. What was the reason you did not want your family member or significant other to participate in the ULTRA study? You do not need to answer this question. If you do not answer this question, there will be no consequences for the future treatment of your family member or significant other.
o Me, my family member or significant other never participate in scientific medical research
o It takes too much time
o Privacy reasons
o The way of asking consent and the procedures for the ULTRA study
o Other reason(s), namely: ………………………………………………………………………………………………………………………………………

2. As was explained in the information letter, the way of asking consent and the procedures for the ULTRA study are different from other studies. Only after administration of the study medication to your family member or significant other, you received an explanation about the study. At the time of explanation, you were asked for consent to use the data for research purposes. If your family member or significant other did not receive the study medication, the explanation about the study and asking for consent also took place after drawing of lots. This was done because the treatment investigated in the ULTRA study has to be administered as soon as possible after the brain hemorrhage. Do you agree with the way of asking consent and the procedures for the ULTRA study?
o Yes. You can continue with question 4.
o No. You can continue with question 3.

3. Why do you not agree with the way of asking consent and the procedures for the ULTRA study? If you would like to, you can check multiple answers.
o Your opinion is that you should receive an explanation about the study before you consent for your family member or significant other to participate in the study. This should also be done if this means it is practically not possible to carry out the study and that the possible benefit to your family member or significant other no longer applies
o Other reason(s), namely:

……………………………………………………………………………………………………………………………………….…………………………………………………………………………………………………………………………………………….…………………………………………………………………………………………………………………………………

4. Did the way of asking consent and the procedures for the ULTRA study change your trust in physicians?
o I have more trust in physicians
o My trust in physicians remained the same
o My trust in physicians decreased

5. Did the way of asking consent and the procedures for the ULTRA study change your trust in scientific research?
o I have more trust in scientific research
o My trust in scientific research remained the same
o My trust in scientific research decreased

6. Which way of asking consent and which procedures do you prefer for the ULTRA study?
o An explanation about the study is provided and afterwards consent for participation in the study is asked. Treatment with study medication can only begin when consent is provided. Because of the possibility of a cooling-off period, the study medication will be administered at a later point in time, while the risk of a new bleeding is highest just after the first bleeding.
o Consent is asked for participation in a scientific study without any additional explanation about what the study exactly entails. The explanation about the study will be given after the study has been completed.
o Lots are drawn and if the outcome is treatment, the study medication will be administered. Only after administration of the study medication, you will receive an explanation about the study and are asked for consent to continue participation.
o It does not matter to you which way of consent and procedures are used for the ULTRA study.

7. Did the way of asking consent and the procedures for the ULTRA study change your opinion to participate in future scientific research?
o Yes, I am more willing to participate in future scientific research
o Yes, I am less willing to participate in future scientific research
o No, my willingness to participate in future scientific research remained the same

8. Would you like us to inform you about the results (at group level) of this survey?
o Yes
o No

Thank you for filling out this survey. For questions or remarks, you can contact the researchers (Appendix II).

Survey for physicians of centers that agreed to participate in the ULTRA trial

**Survey about the ULTRA study**

1. For most studies it is common that the patient/proxy is informed about the study before consent is asked (=informed consent). For the ULTRA study, a deferred consent procedure is used because it was otherwise not possible to investigate if treatment with tranexamic acid is effective. Do you think the patient/proxy considers deferral of consent in the ULTRA trial ethically acceptable?
o Acceptable
o Not acceptable

2. Do you consider the consent procedure used in the ULTRA study ethically acceptable?
o Acceptable. You can continue with question 4.
o Not acceptable. You can continue with question 3.

3. If you consider the consent procedure in the ULTRA study to be non-acceptable, can you specify why? You can check multiple answers.
o Patient/proxy should be informed about the content, aim, and risks of the study before consent is asked (=informed consent). Also if this means it is practically not possible to carry out the study and that the possible benefit for the patient no longer applies.
o Other reason(s), namely:
……………………………………………………………………………………………………………………………………….…………………………………………………………………………………………………………………………………………….…………………………………………………………………………………………………………………………………

4. Which consent procedure would you prefer for the ULTRA study?
o Informed consent: Patient/proxy will be informed about the content, aim, and risks of the study before consent is asked and treatment initiated. Because of the possibility of a cooling-off period, the study medication will be administered at a later point in time, while the risk of a new bleeding is highest just after the first bleeding.
o Modified consent: Patient/proxy gives consent for participation in scientific research, but without information about the content, aim, and risks of the study. If the study has been completed, the patient/proxy will be informed about the content, aim, and risks of the study.
o Deferred consent: Lots are drawn and if the outcome is treatment, the study medication will be administered. Afterwards, information about the content, aim, and risks of the study will be provided and the patient or proxy is asked for consent to continue participation in the study.
o It does not matter to you which way of consent and procedures are used for the ULTRA study.

5. Would you like us to inform you about the results (at group level) of this survey?
o Yes
o No

Thank you for filling out this survey. For questions or remarks, you can contact the researchers (Appendix II).

Survey for physicians of centers that declined to participate in the ULTRA trial

**Survey about the ULTRA study**

1. What was the reason your hospital did not want to participate?
o The amount of time required
o The consent procedure
o No financial compensation for included patients
o Other reason(s), namely: ………………………………………………………………………………………………………………………………………

2. For most studies it is common that the patient/proxy is informed about the study before consent is asked (=informed consent). For the ULTRA study, a deferred consent procedure is used because it was otherwise not possible to investigate if treatment with tranexamic acid is effective. Do you think the patient/proxy considers deferral of consent in the ULTRA trial ethically acceptable?
o Acceptable
o Not acceptable

3. Do you consider the consent procedure used in the ULTRA study ethically acceptable?

o Acceptable. You can continue with question 5.

o Not acceptable. You can continue with question 4.

4. If you consider the consent procedure in the ULTRA study to be non-acceptable, can you specify why? You can check multiple answers.
o Patient/proxy should be informed about the content, aim, and risks of the study before consent is asked (=informed consent). Also if this means it is practically not possible to carry out the study and that the possible benefit for the patient no longer applies.
o Other reason(s), namely:

……………………………………………………………………………………………………………………………………….…………………………………………………………………………………………………………………………………………….…………………………………………………………………………………………………………………………………

5. Which consent procedure would you prefer for the ULTRA study?
o Informed consent: Patient/proxy will be informed about the content, aim, and risks of the study before consent is asked and treatment initiated. Because of the possibility of a cooling-off period, the study medication will be administered at a later point in time, while the risk of a new bleeding is highest just after the first bleeding.
o Modified consent: Patient/proxy gives consent for participation in scientific research, but without information about the content, aim, and risks of the study. If the study has been completed, the patient/proxy will be informed about the content, aim, and risks of the study.
o Deferred consent: Lots are drawn and if the outcome is treatment, the study medication will be administered. Afterwards, information about the content, aim, and risks of the study will be provided and the patient or proxy is asked for consent to continue participation in the study.
o It does not matter to you which way of consent and procedures are used for the ULTRA study.

6. Would you like us to inform you about the results (at group level) of this survey?
o Yes
o No

Thank you for filling out this survey. For questions or remarks, you can contact the researchers (Appendix II).

**Table A1.** Survey results patients or proxies

| **Survey questions** | **Patients**  **(%)**  **N=45** | **Proxies**  **(%)**  **N=44** |
| --- | --- | --- |
| Do you agree with the use of deferred consent in the ULTRA trial?   - Yes - No - Question not answered | 41/45 (91)  0/45 (0)  4/45 (9) | 41/44 (93)  1/44 (2)  2/44 (5) |
| Can you explain why you do not approve the way of asking consent and the procedures for the ULTRA study?   - Informed consent should be asked before trial inclusion - Other reason(s) | N/A  N/A | 0/1 (0)  1/1 (100) |
| Could you specify the reason why you did not want to participate in the ULTRA study?   - I never participate in scientific research - It takes too much time - Privacy reasons - The way of asking consent and the procedures for the ULTRA study - Other reason(s) | 0/2 (0)  1/2 (50)  0/2 (0)  0/2 (0)  1/2 (50) | 0/1 (0)  0/1 (0)  0/1 (0)  0/1 (0)  1/1 (100) |
| Did the use of deferred consent change your trust in physicians?   - Increase in trust - Remained the same - Decrease in trust | 2/45 (4)  43/45 (96)  0/45 (0) | 7/44 (16)  37/44 (84)  0/44 (0) |
| Did the use of deferred consent change your trust in scientific research?   - Increase in trust - Remained the same - Decrease in trust | 8/45 (18)  37/45 (82)  0/45 (0) | 12/44 (27)  32/44 (73)  0/44 (0) |
| Which consent procedure do you prefer for the ULTRA trial?   - Deferred consent - Consent for research without explaining its content - Informed consent - No preference - Question not answered | 17/45 (38)  3/45 (7)  7/45 (16)  16/45 (36)  2/45 (4) | 14/44 (32)  5/44 (11)  8/44 (18)  16/44 (36)  1/44 (2) |
| Did the use of deferred consent in the ULTRA trial change your willingness to participate in future research?   - Increase in willingness - Remained the same - Decrease in willingness - Question not answered | 7/45 (16)  36/45 (80)  1/45 (2)  1/45 (2) | 18/44 (41)  23/44 (52)  3/44 (7)  0/44 (0) |

Legends: ULTRA = ULtra-early TRAnexamic acid after subarachnoid hemorrhage; N/A = not applicable.
